# Nanocurcumin: A Promising Candidate for Therapeutic Applications

**DOI:** 10.3389/fphar.2020.00487

**Published:** 2020-05-01

**Authors:** Adhimoolam Karthikeyan, Natesan Senthil, Taesun Min

**Affiliations:** ^1^Subtropical Horticulture Research Institute, Jeju National University, Jeju, South Korea; ^2^Department of Plant Molecular Biology and Bioinformatics, Center for Plant Molecular Biology and Biotechnology, Tamil Nadu Agricultural University, Coimbatore, India; ^3^Faculty of Biotechnology, College of Applied Life Science, Sustainable Agriculture Research Institute (SARI) and Jeju International Animal Research Center (JIA), Jeju National University, Jeju, South Korea

**Keywords:** curcumin, *Curcuma longa*, diferuloylmethane, nanoformulation, turmeric

## Abstract

*Curcuma longa* is an important medicinal plant and a spice in Asia. Curcumin (diferuloylmethane) is a hydrophobic bioactive ingredient found in a rhizome of the *C. longa*. It has drawn immense attention in recent years for its variety of biological and pharmacological action. However, its low water solubility, poor bioavailability, and rapid metabolism represent major drawbacks for its successful therapeutic applications. Hence, researchers have attempted to enhance the biological and pharmacological activity of curcumin and overcome its drawbacks by efficient delivery systems, particularly nanoencapsulation. Research efforts so far and data from the available literature have shown a satisfactory potential of nanorange formulations of curcumin (Nanocurcumin), it increases all the biological and pharmacological benefits of curcumin, which was not significantly possible earlier. For the synthesis of nanocurcumin, an array of techniques has been developed and each technique has its own advantages and individual characteristics. The two most popular and effective techniques are ionic gelation and antisolvent precipitation. So far, many curcumin nanoformulations have been developed to enhance curcumin delivery, thereby overcoming the low therapeutic eﬀects. However, most of the nanoformulation of curcumin remained at the concept level evidence, thus, several questions and challenges still exist to recommend the nanocurcumin as a promising candidate for therapeutic applications. In this review, we discuss the different curcumin nanoformulation and nanocurcumin implications for different therapeutic applications as well as the status of ongoing clinical trials and patents. We also discuss the research gap and future research directions needed to propose curcumin as a promising therapeutic candidate.

## Introduction

*Curcuma longa* commonly referred to as turmeric is an ancient perennial herb belonging to the family Zingiberaceae and native to India. *Curcuma* has developed by incessant cross-breeding and selection. To date, over 100 known species are reported in the species of *Curcuma* (Esatbeyoglu et al., 2012). Besides, the widespread *Curcuma longa* (syn. *Curcuma domestica*), *Curcuma aromatica*, and *Curcuma xanthorrhiza* are other common species (Itokawa et al., 2008). It is grown in tropical and subtropical areas of the world, extensively cultivated in Asian countries, viz., India, Burma, Bangladesh, China, Indonesia, Japan, Taiwan, Thailand, and Vietnam (Chattopadhyay et al., 2004; Damalas, 2011). **Curcuma** species exhibit inter and intraspecific differences in the biologically active principles combined with morphological differences in the above-ground vegetative and floral characteristics and the under-ground rhizome characteristics (Sasikumar, 2005). **Curcuma** has a strong relationship with the socio-cultural life of the people of Asia, using it as a medicine, nutritional spice, and food preservative.

Curcumin is an important bioactive ingredient isolated from the rhizomes of *C. longa* (Tayyem et al., 2006; Heger et al., 2014). In the middle of the 20^th^ century, researchers described the biological features of curcumin. Three sovereign research teams identified various features of curcumin in the 1970s, including cholesterol-lowering (Patil and Srinivasan, 1971), antidiabetic (Srinivasan, 1972), anti-inflammatory (Srimal and Dhawan, 1973), and anti-oxidant (Sharma, 1976) activities. Curcumin has been shown to control various signaling molecules at the molecular level based on the target and cell background. It can trigger up or down-regulation. Thus, it acts on multiple targets in cellular pathways creating an agent that able to complete multiple actions (Paulraj et al., 2019). In human, the biological activity of curcumin relies on its bioavailability. Studies of bioavailability have detailed the amount and concentration at which curcumin is engrossed, occurs in the plasma, and entering its target location.

In the recent three decades, researchers have worked on curcumin for its various functional and biological features viz., anti-inflammatory, anti-oxidant, anti-mutagenic, antimicrobial activity, anti-tumoral, wound healing, and antiangiogenesis effects (Mahady et al., 2002; Aggarwal and Harikumar, 2009; Akbik et al., 2014; Hu et al., 2015; Fernández-Bedmar and Alonso-Moraga, 2016; Da Silva et al., 2018; Imran et al., 2018; Willenbacher et al., 2019). Existing research data provide evidence to support the curcumin’s beneficial effects on different human diseases including cancer (Adiwidjaja et al., 2017), diabetes (Shome et al., 2016), lung and chronic kidney diseases (Gupta et al., 2013; Trujillo et al., 2013), neurological disorders (Aggarwal and Sung, 2009), metabolic disease (Panahi et al., 2016), liver problems (Nabavi et al., 2014), cardiovascular disease (Bhullar et al., 2013), digestive disorders (Debjit Bhowmik et al., 2009), and other inflammatory diseases (Beevers and Huang, 2011). Despite its reported benefits, multiple factors often limit the practical applications of curcumin. For instance, poor water solubility and physicochemical instability, low pharmacokinetics and bioavailability, poor bioactive absorption, rapid metabolization, low penetration and targeting efficacy, sensitivity to alkaline conditions, metal ions heat and light (Flora et al., 2013). However, these obstacles being solved by encapsulating curcumin into nanoformulations (nanocurcumin) (Yallapu et al., 2012a). Integrating curcumin into nanocarriers through various methods is an appropriate and fruitful choice to upsurge the biological activity of curcumin, which increases its bioavailability and solubility, long time circulation, and retention in the body, and overcome physiological barriers of curcumin (Sahu et al., 2008; Das et al., 2010; Li et al., 2013; Bhatia et al., 2016; Fonseca-Santos et al., 2016). Also, it can reduce the unintended toxicity to surrounding normal cells/tissues by diffusing the indent tissues.

So far, many researchers showed the feasibility of using nanoformulation based approaches to improve curcumin application in both *in vitro* and *in vivo* studies that involve the use of liposomes, polymers, conjugates, cyclodextrins, micelles, dendrimers, and nanoparticles (Ghalandarlaki et al., 2014; Naksuriya et al., 2014; Yallapu et al., 2015). Of these, some curcumin nanoformulations have extended clinical studies and applications. Since 2011, more than 1,500 publications related to curcumin nanoparticles were available in the NCBI PubMed database (http://www.ncbi.nlm.nih.gov/sites/entrez, accessed 6^th^ March 2020). In the beginning, many researchers worked mainly to improve bioavailability but later also focused on effective curcumin targeting in the diseased area with peptide mediation, aptamer, and antibody support. Curcumin was encapsulated into poly(lactic-co-glycolic acid) nanoparticles (PLGA NPs) and oral bioavailability was examined. Results showed a nine-fold increase in nanocurcumin over the native curcumin (Shaikh et al., 2009). Experimental data also support that nanoform of curcumin produced an effective result against liver and heart problems (Shimatsu et al., 2012), cancers (Mohanty and Sahoo, 2010), and brain tumors (Lim et al., 2011).

In this review first, we briefly discuss chemistry and molecular targets of curcumin and methods for synthesis of curcumin nanoformulation. In the next section, different curcumin nanoformulations, comparative characteristics of curcumin and nanocurcumin, and nanocurcumin implications in various therapeutic applications are summarized and discussed. In the final section of this review, we discussed the status of ongoing clinical trials and patents, the research gap, and future research directions needed to propose curcumin as a promising therapeutic candidate.

## Chemical Structure and Molecular Targets of Curcumin

**Figure 1:**
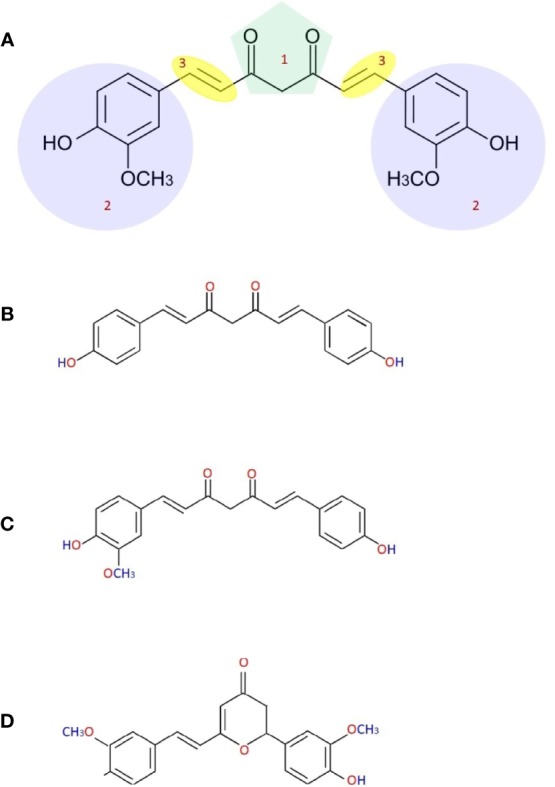
Chemical structure of curcumin **(A)** [(1) b-diketone or keto-enol, (2) phenolic, (3) alkene linker], demethoxycurcumin **(B)**, bisdemethoxycurcumin **(C)** and cyclocurcumin **(D)**.

The probable chemical composition of curcumin was described by many researchers in the eighteenth century. Curcumin’s International Union of Pure and Applied Chemistry (IUPAC) name is (*1E,6E*)-1,7-bis(4-hydroxy-methoxyphenyl)-1,6-heptadiene-3,5-dione (Miłobdzka et al., 1910). Chemical formula and the curcumin’s molecular weight are C_21_H_20_O_6_ and 368.38 g/mol. Curcumin has three chemical substances: two aromatic ring systems of o-methoxy phenolic groups, linked by a seven-carbon connector comprising a α,β-unsaturated β-diketone moiety (Nelson et al., 2017). This chemical structure makes curcumin less soluble in water at acidic and neutral pH but soluble in ethanol, alkali, ketone, methanol, acetic acid, and chloroform, dimethyl sulfoxide (DMSO), and acetone. The melting temperature of curcumin is 176–177°C (Mošovská et al., 2016) and it has various methoxy substitutions in the diferuloylmethane chemical structure (which is responsible for yellow coloration), demethoxycurcumin, bisdemethoxycurcumin, and cyclocurcumin (**Figure 1**) are responsible for many biological and pharmacological differential activities of these compounds (Anand et al., 2008). Somparn et al. reported that diferuloylmethane shows better antioxidant activity than demethoxycurcumin and bisdemethoxycurcumin, and demethoxycurcumin to have a potent antioxidant effect than bisdemethoxycurcumin (Somparn et al., 2007). A well-known antioxidant mechanism that exists in curcumin is transition metal chelation that is linked to the moieties of diketone and o-methoxy phenols. Diferuloylmethane, demethoxycurcumin, and bisdemethoxycurcumin obstruct the hemeoxygenase‐1 and NF‐kB so as are responsible for the structural moieties of α,β‐unsaturated diketone that act as an acceptor for Michael reaction (Jeong et al., 2009; Rajasekaran, 2011). When the heptadiene moiety is hydrogenated and curcumin is administrated intraperitoneally tetrahydrocurcumin is produced. The antioxidant property of this compound is considerably higher than curcumin with decreased antitumor and anti-inflammatory activities (Somparn et al., 2007; Itokawa et al., 2008).

**Figure 2:**
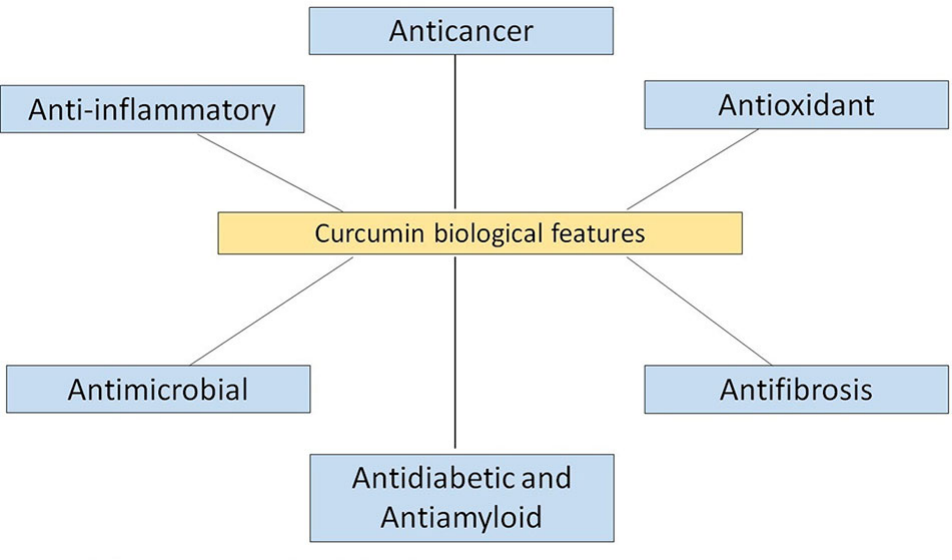
Different functional and biological features of curcumin or nanocurcumin.

**Figure 3:**
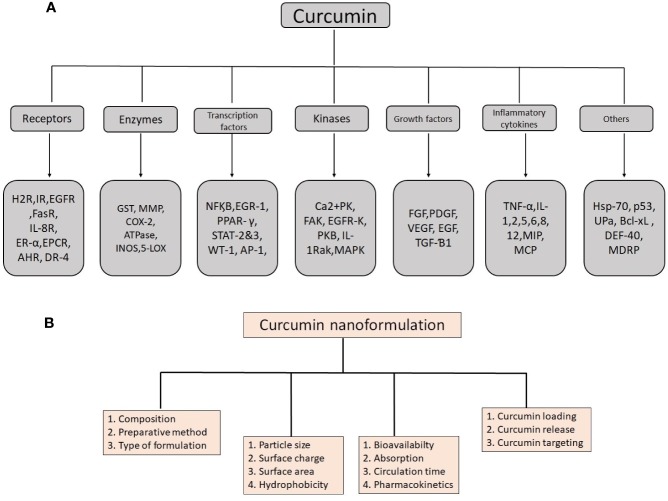
Details of molecular targets mediated by curcumin **(A)** and important physico-chemical properties and their role in biological functions to remember when producing an effective nanoformulation of curcumin **(B)**.

Curcumin contains many valuable biological properties (**Figure 2**) and molecular mechanism of these properties are given in **Table 1**. It is capable to bind and obstruct different proteins, metals, growth factors, transcription factors, receptors, enzymes, and other important biomolecules (Goel et al., 2008) directly and indirectly. So far, many researchers have investigated the molecular targets of curcumin and identified the direct targets include metal ions, inflammatory molecules, protein kinases/reductases, proteasomes, DNA methyltransferase 1, carrier proteins, and cell survival proteins. The indirect targets comprise enzymes, transcription factors, adhesion molecules, mediators of inflammation, receptors, growth factors, proteins regulating the cell cycle, and proteins for cell survival (Gupta et al., 2011). Several molecular targets mediated by curcumin are summarized in **Figure 3**. Curcumin is a pleiotropic molecule that can interact with several inflammatory-related molecular targets (Zhou et al., 2011). It controls the inflammatory response by decreasing the activity of inducible nitric oxide synthase (iNOS), lipoxygenase (LOX), phospholipases A2 (PLA2s), and cyclooxygenase-2 (COX-2) enzyme pathway that obstructs the prostaglandin synthesis and pro-inflammatory leukotrienes and essential inflammatory response mediators (Farhood et al., 2019). Curcumin inflammatory response is closely related to the arachidonic acid pathway for eicosanoid biosynthesis, which produces a host of reactive lipid products such as prostaglandins, thromboxanes, leukotrienes, and prostacyclins. Curcumin downregulated the activities of LOX and COX-2 at the transcriptional level and through inhibition of the post-translational enzyme that leads to a reduction in arachidonic acid metabolism (Huang et al., 1991; Zhang et al., 1999; Rao, 2007). Also, curcumin has been shown to obstruct the biosynthesis of prostaglandin E2 by direct inhibition of the microsomal prostaglandin E2 synthase-1 enzyme (Koeberle et al., 2009). Curcumin’s free-radical scavenging activity also related to its anti-inflammatory properties by reducing the level of oxidative stress that could cause the inflammatory cascade. Lin reported curcumin is stated to have anti‐inflammatory and inhibitory effects on major ROS‐producing enzymes (Lin, 2007). NF‐kB is the major pro-inflammatory transcription factor targeted by curcumin. It is responsible for the genes related to tumor growth. Curcumin has been shown that strong inhibitory activities on NF‐kB activation and expressions of some oncogenes.

**Table 1:** Biological properties and their molecular mechanism of curcumin or nanocurcumin.

S. No	Biological properties	Molecular mechanism	References
1	Anti-inflammatory	Curcumin control the inflammatory response through decreasing the activity of cyclooxygenase-2 (COX-2), lipoxygenase (LOX), phospholipases A2 (PLA2s) and inducible nitric oxide synthase (iNOS) enzymes pathway that obstructs the prostaglandin synthesis and pro-inflammatory leukotrienes and essential inflammatory response mediators	Shakeri and Boskabady, 2017; Yuan et al., 2018; Farhood et al., 2019; Kumari et al., 2019; Lee et al., 2020
2	Anticancer	STAT3 and NF-κB signaling pathways play major role in cancer growth, curcumin effectively obstruct the activity of STAT3 and NF-κB. Besides, curcumin obstructs cancer formation, migration, and invasion by control the expression of Sp-1 and its housekeeping genes.	Vallianou et al., 2015; Buhrmann et al., 2019; Chung et al., 2019; Wong et al., 2019; Khan et al., 2020
3	Antiamyloid	Curcumin regulates amyloid beta (Aβ) metabolism and inhibits Aβ aggregation and as well as disaggregates to form fibrillar Aβ16, Aβ40, and Aβ42 many ways to produce strong anti‐amyloidogenic effects.	Rao et al., 2015; Brahmkhatri et al., 2018; Hotsumi et al., 2019
4	Antioxidant	Curcumin can ability to scavenge free radicals (i.e., ROS and RNS and also modulate the enzymes (GSH, catalase, and SOD) activity to neutralize the free radicals. Besides, curcumin also obstructs ROS-producing enzymes (i.e., lipoxygenase/cyclooxygenase).	Jagetia and Rajanikant, 2015; Lourestanpour et al., 2017; Abrahams et al., 2019; Fakhri et al., 2019
5	Antimicrobial	The potential mechanism underlying curcumin antimicrobial activity related to FtsZ that is vital cell division initiating protein.	Groundwater et al., 2017; Da Silva et al., 2018; Rai et al., 2020
6	Antifibrosis	Curcumin prevent migration, collagen production, and proliferation abilities of fibroblast through modulating the expression of transforming growth factor (TGF)-β and angiotensin signaling (Ang).	Sun et al., 2017; Chen X. et al., 2020
7	Antidiabetic	The mechanism through which curcumin suppresses advanced glycation end products (AGEs) formation is suggested to involve the suppression of AGE receptor (RAGE) expression through the activation of peroxisome proliferator-activated receptor gamma (PPARγ) activity and increase in glutathione synthesis. Increasing secretion of insulin from pancreatic cells, reduces insulin resistance.	Wojcik et al., 2018; Gutierres et al., 2019; Den Hartogh et al., 2020

Many studies showed that curcumin act as a blocking agent and obstructing a preliminary stage of cancer. It also has activity as a suppressive agent for inhibiting the proliferation of malignant cells during carcinogenesis elevation and development. Curcumin’s mechanisms for its anticancer activities are inclusive and varied, affecting multiple stages of control in cellular growth and apoptosis process. Due to the extensive activities and multiple targets of curcumin on the cellular growth regulatory processes, it has great potential as a chemotherapeutic agent for human cancers (Kwon, 2014). Moreover, curcumin action on many signaling proteins, oncogenes, and transcription factors, it also involves the course of tumorigenesis, growth, and metastasis at different stages of carcinogenesis from the early effects that cause DNA mutations (Wilken et al., 2011). Curcumin arrests tumor growth by obstructing some key signal transduction pathways (Shehzad et al., 2010). Transcription factors, namely activating protein-1 (AP-1), signal transducer, and activator of transcription (STAT) proteins associated with tumorigenesis negatively regulated by curcumin. It triggers apoptosis cell death by preventing the loss of N‐CoR protein that is misfolded and ubiquitin‐proteasome pathway damage (Ng et al., 2011). Another main target of curcumin is protein kinases. Epidermal growth factor receptor and the mitogen-activated protein kinase activity in pancreatic and lung adenocarcinoma cells were downregulated by curcumin. Research to date suggests that antiamyloid activity mechanism of curcumin linked with the reduction of amyloid-β-protein (Aβ) aggregation, and Aβ-induced inflammation, as well as the activities of β-secretase and acetylcholinesterase (Hotsumi et al., 2019).

Curcumin possesses many forms of functional groups (diketo group, carbon-carbon double bonds, and phenyl rings) in its structure. Thus, compared to other antioxidants, curcumin is a unique and potent antioxidant agent, though the knowledge of this antioxidant mechanism remains questionable. No thorough knowledge has been available until now on whether the phenol or the CH2 moiety of the heptadienone branch is responsible for the antioxidant activity of curcumin. Jovanovic et al. identified curcumin as a superb H-atom donor by giving the H-atom in acidic and neutral aqueous and acetonitrile solutions from the central methylenic group rather than from the phenolic group (Jovanovic et al., 1999). Contrarily, Barclay et al. described that curcumin is a classical phenolic antioxidant, which breaks the chain and gives the phenolic group H-atoms (Barclay et al., 2000). Theoretical calculations by the density functional theory (DFT) showed that the enol type of curcumin is much stable than the diketo form and that the bond dissociation enthalpy (BDE) of the phenolic O:H bond is pointedly less than the BDE of the central O:H bond, confirming that abstraction of the hydrogen atom receipts the place within the phenolic group. Besides, it suggests that the relative contribution of the phenolic group and the central methylenic group on the antioxidant activity based on the activity of aggressive radical and the reaction medium (Menon and Sudheer, 2007). Curcumin has been shown to inhibit the progress of fibrosis by reducing the cytokines and chemokine genes expression, these genes are directly related to the fibrosis and the initiation of apoptosis in stellate cells of affected organs (Gorabi et al., 2019). The antimicrobial activity mechanism of curcumin is renowned, and its antimicrobial mechanisms related to the interaction with the FtsZ protein. FtsZ is a cell division initiation protein that exists in most of the prokaryotic species and plays a major role in the division of chloroplasts and mitochondria in some eukaryotes. The bacterial cytoskeleton is required for growth and cell division, FtsZ protein is involved in the division of bacterial cells, and is the first protein to appear at the impending site of division (Da Silva et al., 2018).

## Techniques for Synthesis of Curcumin Nanoformulation

An array of techniques has been developed for the synthesis of nanocurcumin. The most common techniques include nanoprecipitation, single emulsion, microemulsion, spray drying, emulsion polymerization, solvent evaporation, antisolvent precipitation, ultra-sonication, coacervation technique, ionic gelation, wet milling, solid dispersion, thin-film hydration, and Fessi method. Each technique has own advantages and individual characteristics reviewed by many researchers (Rai et al., 2015; Rajalakshmi and Dhivya, 2018). Here, we discuss ionic gelation and antisolvent precipitation, which are the two most efficient and superior techniques. With in-depth review of the published works of the literature suggest that ionic gelation and the antisolvent precipitation-based synthesis of curcumin nanoparticles showed better solubility and stability compared to other techniques. Ionic gelation technique is based on the capability of polymers to crosslink in the presence of counter ions (Giri et al., 2013). This technique emerged as one of the most promising systems for preparing natural polymers (chitosan/alginate) that are non-toxic, biocompatible, and biodegradable (Giri, 2016). Several studies have been detailed the potential and use of natural polymer (chitosan/alginate) nanoparticles for oral delivery of curcumin based on this method (Bhunchu et al., 2015; Bhunchu et al., 2016). Das et al. reported nanoformulation of curcumin tripolymeric composite (alginate, chitosan, and pluronic) developed using ionic gelation technique and their delivery to cancer cells (Das et al., 2010). Akhtar et al. prepared curcumin bound chitosan nanoparticles and demonstrated the feasibility of using this technique to improve the antimalarial activity in mice along with better metabolic stability and bioavailability (Akhtar et al., 2012). Antisolvent precipitation is another widely used technique to prepare the curcumin nanoparticles and the efficacy of this technique depends on the time interval, temperature, and stirring speed. Many studies reported that the antisolvent precipitation technique is promising and cost-effective technique. It provides better solubility and stability of the curcumin nanoparticles. This simple operates technique is easy to apply for the industrial production of drug nanoparticles (Kakran et al., 2012; Yadav and Kumar, 2014).

## Curcumin Nanoformulations

Over the past several years, many curcumin nanoformulations (**Table 2**) have been developed. Most of them focusing on improving curcumin’s bioavailability and solubility and shielding curcumin from hydrolysis inactivation. Some formulations are targeted for longtime circulation and retention in the body, while reminders have focused on cellular delivery and intracellular release mechanisms. Several curcumin nanoformulations created a great impact on pharmaceutical applications and confirmed to have useful in the diagnosis of various human diseases. They are outlined and discussed here.

**Table 2:** Summary of curcumin nanoformulations and their therapeutic role.

S. No	Curcumin nanoformulation	Description	Models used	Major outcomes	References
1	Liposomes	Liposomes are a spherical vesicle consisted of single or multiple phospholipid bilayers surrounding aqueous units that very closely resemble the cell membrane structure. It solubilizes curcumin in the phospholipidic bilayer and allows curcumin to be distributed in aqueous medium and increases the effect of curcumin.	Malaria, melanoma, renal ischemia, colorectal cancer, and lung cancer	Increased the antimalarial and antimelanoma effects, greater encapsulation efficiency, excellent bioactivity, and anticancer activity	Basnet et al., 2012; Rogers et al., 2012; Tefas et al., 2017; Sesarman et al., 2018; Chen et al., 2019; Huang et al., 2019; Reddy et al., 2019; Vetha et al., 2019
2	Polymers	Polymers are another widely used effective drug delivery system for curcumin. It can able to improve the oral bioavailability and solubility of curcumin.	Wound healing and colorectal cancer	Exhibited strong wound healing and long blood circulation, suppression of tumor growth, higher growth inhibition in cancer cells than free curcumin, and increased the cellular uptake and better anticancer activity	Li et al., 2012; Anitha et al., 2014; Chaurasia et al., 2016; Xie et al., 2017; Moballegh Nasery et al., 2020
3	Gold nanoparticles	Gold nanoparticles have own unique physical and chemical properties and various surface functionalities. It offers versatalite platform in drug delivery (curcumin)	Prostate and colorectal cancer cells	Improved antioxidant activity, extended blood circulation, better solubility and stability, enhanced biocompatibility, and considerable anticancer activity	Singh et al., 2013b; Rejinold et al., 2015; Nambiar et al., 2018; Elbialy et al., 2019
4	Magnetic nanoparticles	Magnetic nanoparticles used for multiple purposes including drug delivery (curcumin), hyperthermia, and quality imaging	Cancer and inflammatory cells	Improved cellular uptake, potent targeting capability of curcumin, magnetic resonance imaging, effective protection against inflammatory agent, controlled curcumin delivery, excellent bio-compatibility, and anticancer activity	Yallapu et al., 2012b; Bhandari et al., 2016; Saikia et al., 2017; Aeineh et al., 2018; Ayubi et al., 2019
5	Solid lipid nanoparticles (SLNs)	SLNs possess a lipid core matrix that can solubilize drug (curcumin) and the lipid core is steadied through emulsifiers. Normally SLN is spherical in shape.	Allergy, colitis and cerebral ischemia, and breast cancer lines	Extended circulation of blood, increased anti-inflammatory effects, targeted and enhanced drug release in brain, and better anticancer activity	Yadav et al., 2009; Kakkar et al., 2011; Wang et al., 2012; Kakkar et al., 2013; Bhatt et al., 2018; Wang W. et al., 2018; Fathy Abd-Ellatef et al., 2020
6	Conjugates	The complex formed from the joining together of two or more molecules, especially by covalent bond is referred as conjugates. Curcumin conjugation with small molecules and hydrophilic polymers increase its solubility and oral bioavailability	Fibroblast cells, breast cancer, and amyloid fragments	Increased the solubility, stability and bioavailability, strong anti-cancer activity, higher stability and bioavailability, and anti-amyloid effects	Manju and Sreenivasan, 2011; Singh et al., 2013a; Brahmkhatri et al., 2018
7	Cyclodextrins	Cyclodextrins are the bucket shaped oligosaccharides and well known solubilizing and stabilizing agent. It can solubilize the curcumin in a lipophilic cavity, and the outer hydrophilic surface assists in greater dispersion of the formulation.	Bowel disease, lung, pancreatic, breast, colorectal cancer, and prostate cancer cells	Developed bioavailability and increased solubility, improved antiproliferation, anticancer and anti-inflammatory effects, increased the solubility, and formulated as eye drops.	Yallapu et al., 2010; Yadav et al., 2012; Dandawate et al., 2012; Abruzzo et al., 2016; Ntoutoume et al., 2016; Nabih Maria et al., 2017; Guo, 2019
8	Solid dispersions	Solid dispersions are referred as one or more active component in an appropriate matrix. It can improve the bioavailability of poor water soluble drugs like curcumin.	Breast tumor, rat paw edema, and wound healing	Prolonged survival, anti-tumor and anti-metastasis activity and prolonged survival, enhanced stability, bioavailability and anti-inflammatory, anti-bacterial and improvement of vaginal wound healing	Liu et al., 2013; Teixeira et al., 2016; Silva et al., 2019; Zhang et al., 2019
9	Micelles	Micelles (20–100 nm) are normally colloidal dispersions made from amphiphilic molecule. It assist better solubilization and targeted delivery to curcumin.	Lung tumor and colorectal cancer	Bioavailability and solubility improved, prolonged life, targeted drug delivery, great chemical stability, and better antitumor and anticancer effects	Adhikary et al., 2010; Podaralla et al., 2012; Raveendran et al., 2013; Yang et al., 2015; Chang et al., 2016; Javadi et al., 2018; Chen S. et al., 2020
10	Nanospheres	Nanospheres are known as solid matrix particles where in the main component (drug) is mixed, but microcapsule contains internal core and outer polymeric shell.	*Escherichia coli*, *Staphylococcus aureus*, *Vibrio vulnificus*, and *Candida albicans*. Breast cancer cells, melanoma cells, and Alzheimer’s	Exhibited strong antimicrobial and anti-cancer effects, effective target delivery and anti-amyloid effect	Arunraj et al., 2014; Liang et al., 2017; Huo et al., 2019; Masoule et al., 2019; Kim et al., 2020
11	Nanogels	A nanogel is a nanoparticle composed of a hydrogel synthesized by either physical or chemical cross-linking of polymers under controlled conditions. Cross linked structure of nanogels offer a strong base for drug storage and release. It is a possible technique to prepare and release active types of drugs like curcumin to cells for remaining activity, improving stability, and prevent drug immunogenicity	Pancreatic cancer, colorectal cancer and skin cancer cells	Targeted and controlled drug release, prolong circulation, enhanced bio availability, and better anticancer activity	Mangalathillam et al., 2012; Wei et al., 2013; Madhusudana Rao et al., 2015; Amanlou et al., 2019; Wang et al., 2019; Priya et al., 2020
12	Nanodisks	Nanodisks are disk-shaped bilayers, apolipoprotein-stabilized and self-assembled. They boost the solubility and targeted release of curcumin	Mantle cell lymphoma	Improved biological activity and apotopsis to mantle cell lymphoma and anticancer activity	Ghosh et al., 2011; Singh et al., 2011; Subramani et al., 2017

### Liposomes

Liposomes are a spherical vesicle comprised of single or multiple phospholipid bilayers surrounding aqueous units that very closely resemble the cell membrane structure (Faraji and Wipf, 2009). Both *in vitro* and *in vivo* conditions, liposomes are ideal delivery systems for biologically active substances. Liposomes have many advantages such as high biocompatibility and biodegradability, high stability, low toxicity, better solubility, targeting specific cells, controlled distribution, flexibility, and easy preparation (Moballegh Nasery et al., 2020). Thus, liposomes are strong drug-carrier system to date and preferred by researchers. The diameter of the liposome ranges between 25 to 2.5 mm. The vesicle size is an important factor for deciding the circulation time of liposomes and the quantity of drug capsulation in liposomes is influenced by both size and number of bilayers (Akbarzadeh et al., 2013). Many studies have shown that liposome solubilizes curcumin in the phospholipidic bilayer and allows curcumin to be distributed over aqueous medium and increases the effect of curcumin (Chang et al., 2018). Moreover, liposomal drugs accumulate mainly in the liver, spleen, lung, bone marrow, or other tissues and organs. This helps to improve the drug therapeutic index and decrease the side effect. Extensive studies showed that liposomal curcumin was the most suitable vehicle to treat various cancer diseases. Dhule et al. showed that liposomal curcumin inhibited the growth of the KHOS OS cell line and MCF-7 breast cancer cell line and exhibited a strong anticancer effect in both *in vitro* and *in vivo* condition (Dhule et al., 2012). Tian et al. studied the antitumor efficiency and the biochemical mechanisms triggered by curcumin liposomes in PC-3 human prostate cancer cells. The survival rate of curcumin-loaded liposomes treated with PC-3 cells was relatively low and time-depend manner compared with free curcumin (Tian et al., 2014). It was also seen that liposomes could promote the absorption of curcumin into the cell, and the duration of cell fluorescence intensity was higher and longer than the control group. Tefas et al. prepared the liposomes coencapsulating doxorubicin and curcumin, it reduced the cell proliferation in C26 murine colon cancer and showed better cytotoxic activity than its free form (Tefas et al., 2017). Similarly, liposomes co-encapsulating curcumin and resveratrol showed a lower particle size, polydispersity index, and high encapsulation efficiency (Huang et al., 2019). Recently, a combination of curcumin liposome nanocarriers (LIP-CUR) and blue light-emitting diode (BLED) induced photodynamic therapy (BLED-PDT) produced excellent bioactivity and anticancer activity (Vetha et al., 2019). Collectively, the results revealed that the liposomes could be a better carrier for curcumin.

### Nanoparticles

Nanoparticles are particles of approximately 1–100 nm in diameter possess unique physical, chemical, and biological properties that can be useful for drug delivery (Biswas et al., 2014). Nanoparticles are 1,000 times smaller than the average human body cell and consist of materials engineered at the atomic or molecular level. They are also suitable for both controlled and targeted drug delivery systems (Rudramurthy et al., 2016). Encapsulating drugs inside nanoparticles can enhance the pharmacokinetics and solubility of drugs, provide targeted delivery and controlled release of drugs. So far, polymer, solid lipid, magnetic, gold, and albumin-based nanoparticles are extensively used to improve the curcumin therapeutic applications.

Polymeric nanoparticles have the advantage of being small and biocompatible, thus being able to circulate a long time in the blood circulation (Ferrari et al., 2018). Many natural and synthetic polymers include N-isopropylacrylamide (NIPAAM), polyvinyl alcohol (PVA), poly(lactic-co-glycolic acid) (PLGA), polyethylene glycol monoacrylate [NIPAAM (VP/PEG A)], N-vinyl-2-pyrrolidone, silk fibroin, hydrophobically modified starch, and chitosan have been identified and utilized for synthesis of curcumin nanoparticles (Shome et al., 2016). Chang et al. studied the molecular mechanisms activated by curcumin loaded-PLGA nanoparticles in CAL27 cisplatin-resistant cancer cells (CAR cells). Experimental data suggested that curcumin loaded-PLGA nanoparticles controlled the activity of multiple drug resistance protein 1 (MDR1) and the development of reactive oxygen species (ROS) in CAR cells by activating the intrinsic apoptotic pathway. Besides, curcumin-loaded PLGA nanoparticle is more effective against the treatment of CAR cells along with enhanced bioactivity at *in vitro* condition and better bioavailability at **in vivo** condition compare to the native curcumin (Chang et al., 2013). In another study, curcumin loaded polymeric nanoparticles using Eudragit R E100 cationic copolymer exhibited great binding and cellular uptake of polymeric nanoparticles, therefore increasing cytotoxic activity. Taken together, this nanoparticle formulation suppressed tumor growth and reported a 19-fold higher growth inhibition of Colon-26 cells than curcumin alone (Chaurasia et al., 2016). Also, curcumin silk fibroin (CUR-SF) nanoparticles provided a more stable delivery to colon cancer cells and produced a strong anticancer effect than it’s freeform in HCT116 cells. This study concludes controlled release of CUR-SF can able to improve a curcumin cellular uptake into cancer cells and reduce the cytotoxicity to normal cells (Xie et al., 2017).

Solid lipid nanoparticles are the colloidal submicron particles formed through natural or synthetic lipids dispersed in aqueous surfactants or water. They are easily scalable, stable, and biocompatible drug delivery systems with a high drug to lipid ratio which also improves the solubility of poorly soluble drugs (Bhatt et al., 2018). It has been shown that solid lipid curcumin nanoparticles enhanced the solubility over native curcumin and reduced that activity of the lipopolysaccharide (LPS)-induced pro-inflammatory mediators NO, PGE_2_, and interleukin (IL)-6 by obstructing the activation of NF-*κ*B (Nahar et al., 2015). Sun et al. experimental results indicated that curcumin solid lipid nanoparticles (CUR-SLNs) displayed the extended cell uptake and obstruction of growth in cancer cells with improved dispersibility and chemical stability of the drug (Sun et al., 2013). CUR-SLNs were examined for its anticancer activity in breast adenocarcinoma cells (MDA-MB-231). CUR-SLNs showed high solubility and support to drug release in comparison to the native curcumin. Besides, CUR-SLNs induced significantly higher apoptosis in MDA-MB-231 cells. The results suggest CUR-SLNs be useful for cancer treatment (Bhatt et al., 2018). Recently, CURC-SLNs coupled with doxorubicin and used to overcome the Pgp-mediated chemoresistance in triple negative breast cancer cells (TNBC). This formulation appears to be effective and safe due to high biocompatibility and lower toxicity (Fathy Abd-Ellatef et al., 2020).

Magnetic nanoparticles consist of a metal or metallic oxide core that can be functionalized within a polymer or inorganic metal coating. This coating confirms the stability and biocompatibility of the magnetic nanoparticles. They are easily manipulated in size, shape, and chemical properties. Magnetic nanoparticles also have unique physical properties, are biocompatible with the human body, and have a low production cost (Roacho-Pérez et al., 2020). Iron oxide nanoparticle core covered by CD and pluronic polymer (F68) with curcumin showed enhanced uptake in cancer cells. This formulation inhibits the potential of the mitochondrial membrane and produces more ROS than unformulated curcumin. Also, it exhibited a strong anticancer effect together with resonance imaging characteristics and magnetic targeting abilities (Yallapu et al., 2012b). The sustainable delivery of thiolated starch-coated iron oxide nanoparticles containing curcumin displayed significant compatibility of the system in lymphocyte cells. It also caused the cytotoxicity on cancer cell lines due to its higher drug encapsulation, stability, and loading efficiency (Saikia et al., 2017). In another investigation, curcumin loaded Fe3O4-magnetic nanoparticles (MNPs) showed an excellent uptake and helpful for drug releases in tumor tissues. Besides, this formulation accompanied by imaging applications in tumor tissues (Aeineh et al., 2018). Recently, magnetic nanoparticles decorated with PEGylated curcumin (MNP@PEG-Cur) were confirmed as highly biocompatible drug carriers for antitumor medicine (Ayubi et al., 2019).

Albumin is the ideal material and preferable protein carrier for drug delivery due to nontoxic, biocompatible, biodegradable, and high binding capacity with different drugs. Kim et al. study revealed that curcumin-loaded human serum albumin (HSA) nanoparticles (CCM-HSA-NPs) exhibited an enhanced *in vivo* antitumor activity compared to unformulated curcumin in a tumor xenograft animal model, with no toxicity (Kim et al., 2011). Also, experimental data from this study suggested that this formulation is a potential drug delivery system for curcumin in the treatment of cancer. Further, Thadakapally et al. showed that PEG-albumin-curcumin (PAC) nanoparticles have significant anticancer activity in breast cancer lines with stable long circulation and better solubility (Thadakapally et al., 2016). Gold nanoparticles have novel optical and catalytic properties that are non-toxic and biocompatible and drawn significant interest in a variety of applications. In recent years, gold nanoparticles synthesized with plant extracts widely used for the biomedical area (Nambiar et al., 2018). Colloidal stability of these particles keeps the physicochemical properties unchanging. Thus, no changes will occur in the biological activity of the particles. The study conducted using curcumin-encapsulated chitosan-*graft*-poly(*N*-vinyl caprolactam) nanoparticles containing gold nanoparticles (Au-CRC-TRC-NPs) showed targeted delivery of drug and apoptosis to colon cancer cells (Rejinold et al., 2015). In another research, Nambiar et al. synthesized curcumin gold nanoparticles (cur-AuNPs) using cell-culture medium supplemented with or without fetal bovine serum (FBS) and confirmed their anticancer effects in human prostate cancer cells (Nambiar et al., 2018). The gold nanoparticles with curcumin (CWAuNPs) examined for its effects on *in vitro* renal cancer cells. The results confirmed that CWAuNPs was an effective anticancer agent and induced apoptosis in the renal carcinoma cell line A498 (Liu et al., 2019). In a similar manner, curcumin-green synthesized gold nanoparticles (AuNP’s-Cur) evaluated at colon and breast cancer cell lines, HCT-116 and MCF-7 respectively. The study revealed that the AuNP’s-Cur have shown high antiproliferative and apoptotic activity against cancer cells, compared to native curcumin (Elbialy et al., 2019).

### Conjugates

The complex formed from the joining of two or more molecules, especially by the covalent bond is referred to as conjugates. Curcumin conjugation with small molecules and hydrophilic polymers increase its solubility and oral bioavailability. Manju and Sreenivasan reported conjugation of curcumin with hyaluronic acid decreases gold nanoparticles (AuNPs) effects and improves its aqueous solubility and stability (Manju and Sreenivasan, 2011). Singh et al. demonstrated that curcumin conjugates piperic acid and glycine were prepared by esterifying the phenolic hydroxyls of 4 and 4 to increase its bioavailability and trigger apoptosis in MCF-7 and MDA-MB-231 cell lines through a mitochondrion based pathway (Singh et al., 2013a). Similarly, Muangnoi et al. prepared glutaric acid conjugate of curcumin, curcumin-glutaric acid (CurDG) prodrug through ester linkage and tested in mice. It revealed that solubility and antinociceptive properties were increased for CurDG compared to curcumin (Muangnoi et al., 2018). Recently, the gold nanoparticle–PVP–curcumin conjugate (PVP–C–AuNP) found to have obstructed the amyloid Ab (1–6) aggregation with high degree of curcumin bioavailabilty, loading efficiency (80%), and prolonged drug release. This formulation potential to treat Alzheimer’s disease (Brahmkhatri et al., 2018).

### Cyclodextrins

Cyclodextrins [α-, β-, γ-cyclodextrins (CD)] are multi-component hybrid, soluble carrier systems that bear on non-covalent bound drugs. They often used to enhance drug solubility and stability and to deliver drugs in their active form to the cancer cells. Cyclodextrins are bucket-shaped oligosaccharides consisting of six (α-), seven (β-), or eight (γ-) D-glucopyranose units linked through α-1,4-glycosidic bond to form macrocycles (Szejtli, 1998; Ntoutoume et al., 2016). β-CD, γ-CD, and its derivatives were widely used to deliver the drugs due to its low price, relatively easy synthesis and adaptability. Recently, the significance of cyclodextrin in the curcumin delivery system is demonstrated by many researchers (Guo, 2019). Yallapu et al. developed a β-CD mediated curcumin drug delivery system and showed that β-CD-curcumin increased the distribution of curcumin in prostate cancer cells compared to unformulated curcumin and enhanced its therapeutic value (Yallapu et al., 2010). Zhang et al. found that β-cyclodextrin-curcumin (CD15) formulation exhibited high cytotoxicity than normal curcumin through pro-apoptotic and cell cycle arrest activities of lung cancer cells (Zhang et al., 2016). Also, experimental data from this study suggested that CD15 was a potential system for optimizing the delivery of curcumin and its therapeutic efficacy in lung cancer. Nanoparticles were prepared using chitosan, hyaluronic acid, and sulfobutyl-ether-β-cyclodextrin and with or without curcumin and used to treat with intestinal epithelial and colorectal cancer cells. Curcumin nanoparticles showed great encapsulation efficiency and stability. It also decreases the curcumin cytotoxicity in normal intestinal epithelial cells and to reduce cancer cell proliferation (Abruzzo et al., 2016). Further, the water soluble complex of curcumin with cyclodextrins improved solubility and provided the sustained release of drugs in retinitis pigmentosa. The results helped to formulate the eye drops from naturally derived phytochemical (Nabih Maria et al., 2017).

### Solid Dispersions

Molecular dispersion of two various compounds known as a solid dispersion. It is normally a hydrophobic drug (i.e., curcumin in a solid hydrophilic carrier or matrix) (Dhirendra et al., 2009; Flora et al., 2013). To release the drug, solid dispersions are being dissolved as minute colloidal particles of any aqueous media. It diminishes the particle size to nanorange with better wettability increasing the pharmacokinetic properties and oral biodistribution of the drugs. Solid dispersions are produced through fusion-melt, solvent-based methods, and also by combining both the solvent and fusion (hybrid) methods (Tihanyi and Vastag, 2011). Li et al. prepared a curcumin–Eudragit^®^ PO solid dispersion through a solution mixing technique to increase the solubility and stability of curcumin water (Li et al., 2015). Besides, *in vitro* transdermal analysis was performed and confirmed the capability of Cur@EPO as a vehicle to deliver curcumin in medicinal applications. In another study, curcumin-Gelucire^®^50/13 solid dispersion prepared by spray drying showed better solubility (3,600-fold) in water compared with the native curcumin. Besides, the bioavailability and anti-inflammatory activity of curcumin were highly improved by solid dispersion as a consequence of an increased gastrointestinal absorption (Teixeira et al., 2016). Similarly, curcumin solid dispersion-encapsulated temperature-sensitive *in situ* hydrogels (CSDG) effective for treatment for vaginal bacterial infection by stable and sustained release of curcumin (Zhang et al., 2019).

### Micelles

Micelle is referred to as a set of amphiphilic surfactant molecules that spontaneously aggregate in water into a spherical vesicle. It is widely used to deliver poorly water-soluble drugs like curcumin (Rana et al., 2017). Liu et al. used a one-step solid dispersion approach to make curcumin encapsulated polymeric micelles (Cur-M) and studied the effectiveness of Cur-M in a breast tumor model. It was seen that, compared with unformulated curcumin, Cur-M was successful in obstructing the growth of breast tumors and spontaneous pulmonary metastasis of the lungs (Liu et al., 2013). Curcumin-poly(ethylene glycol) methyl ether (MPEG-PCL) micelles solid dispersion enhanced the antiangiogenesis and anti-tumor effect of curcumin. Results from this study also proposed that curcumin micelles may useful in pulmonary carcinoma treatment (Gong et al., 2013). Chang et al. evaluated the outcome of various sizes of curcumin encapsulated micelles on human colon carcinoma cells at *in vitro* condition for their cell uptake, intracellular localization, and cytotoxicity. The results suggest that small sized curcumin loaded micelles have potential to induce better cytotoxicity effect on the human colon carcinoma cells than larger micelles. Explaining thus that drug loading, micelle size and uptake/release kinetics are important considerations for the nanoparticle drug delivery (Chang et al., 2016). Recently, curcumin loaded into the zein-super hydrophilic zwitterionic polymers, poly(sulfobetaine methacrylate) (PSBMA) micelles had much better stability, cellular uptake, cytotoxicity to cancer cells, and pharmacokinetics compared with native curcumin (Chen S. et al., 2020).

### Nanospheres and Microcapsules

Nanospheres are known as solid matrix particles wherein the main component (drug) is mixed, but microcapsule contains the internal core and outer polymeric shell. Arunraj et al. synthesized the surfactant-free curcumin nanospheres (CNSs) and detailed the evidence of CNSs anticancer effect on breast cancer and osteosarcoma cell lines (Arunraj et al., 2014). Smooth and spherical curcumin encapsulated PLGA nanospheres are potential for clinical application in prostate cancer. Cell viability analysis concluded that the curcumin encapsulated nanospheres were capable to exert a further strong activity against cancer cells compared with native curcumin (Mukerjee and Vishwanatha, 2009). Dimethyl curcumin encapsulated PLGA nanospheres (ASC-J9) were evaluated in breast cancer cells. It has been seen that PLGA nanospheres were potential of delivering ASC-J9 intracellularly, most important to arrest the growth of estrogen-dependent MCF-7 cancer cells (Verderio et al., 2014). Curcumin was successfully encapsulated into the poly(ethylene glycol)–poly(lactic acid) (PEG–PLA) nanospheres and delivered to HeLa and MDA-MB-231 cancer cells. This formulation improved curcumin solubility and stability than native curcumin and showed better cytotoxic effects against cancer cells (Liang et al., 2017). To enhance the bioavailability of curcumin, microcapsules containing a solid lipid nanoparticle and mesoporous silica shell were prepared (Kim et al., 2016). It is a promising drug delivery system and more suitable for poorly soluble drugs. Curcumin-polylactic acid (PLA) based microcapsules fabricated through the electrospray method (Mai et al., 2017). The study confirmed the excellent anti-microbial and antioxygenation activity and suggest that the PLA-based electrospray method joint with spherical microcapsules has effective for medicinal applications, particularly drug delivery.

Huo et al. synthesized the selenium nanoparticles (Se NPs) encapsulated poly-lactide-*co*-glycolide (PLGA) nanospheres with curcumin. It decreased the amyloid-β load in Alzheimer’s disease mice, and greatly cured the memory deficiency of the model mice due to effective and targeted drug delivery (Huo et al., 2019)

### Miscellaneous Nanoformulations

Nanogels, nanodisks, yeast cells, and metallo-complexes are other formulations to enhance curcumin’s biological activities. A nanogel is a nanoparticle (10 to 100 nm) composed of a hydrogel synthesized by either physical or chemical cross-linking of polymers under controlled conditions. The cross-linked structure of nanogel offers a strong base for drug storage and release. It is a possible technique to prepare and release active types of drugs to cells for remaining activity, improving stability, and prevent drug immunogenicity (Wang S. et al., 2018). Reeves et al. synthesized and examined a colloidal nanogel carrier system for encapsulation of curcumin to enhance its solubility and cytotoxicity. This curcumin-nanogel formulation was able to kill the tumor cells compared to curcumin alone (Reeves et al., 2015). Dandekar et al. formulated a curcumin loaded hydrogel nanoparticles by combining hydroxypropyl methylcellulose and polyvinyl pyrrolidone and tested the antimalarial activity in mice. It revealed a major action of curcumin-loaded hydrogel nanoparticles over unformulated curcumin (Dandekar et al., 2010). Curcumin loaded into gold nanoparticles-chitosan nanogels showed extent of cellular uptake and better cytotoxic effects on huh7 and MCF7 cell lines compared to native curcumin (Amanlou et al., 2019). In the goal of treating skin cancer, curcumin is delivered as self-assembled capsules with carboxymethyl cellulose and casein nanogels and fabricated with folic acid and casein by layer-by-layer (LbL) technique. The results showed better cellular uptake, cytotoxicity and apoptosis on melanoma cells (MEL-39) (Priya et al., 2020).

Nanodisks are disk-shaped bilayers, apolipoprotein-stabilized and self-assembled. Ghosh et al. first used the nanodisk to boost the solubility and targeted the release of curcumin (Ghosh et al., 2011). Curcumin nanodisk formulations were shown effective strategy to treat MCL or other cancers (Singh et al., 2011). The interaction between curcumin nanodisk and glioblastoma multiforme cells facilitated by ApoE primes to increased curcumin uptake and improved biological activity (Ghosh and Ryan, 2014). Curcumin loading into the *Saccharomyces cerevisiae* cell membrane and other parts were found to be hydrogen-bonded to the cell wall (Paramera et al., 2011a). In another research, Paramera et al. determined the stability of yeast cell–loaded curcumin, it showed that yeast cells restricted the curcumin from environmental factors (i.e., light, humidity, and heat) (Paramera et al., 2011b). Curcumin prepared with Mn (II) and Fe (III) salts exhibited potent activity to Alzheimer’s disease in Swiss albino male rats (Bicer et al., 2018). Palladium (II) complexes with curcumin synthesized had exhibited a strong antitumor effect to MCF-7, HeLa, and A549 tumor cells (Li et al., 2018).

## Comparative Characteristics and Efficacy of Nanocurcumin and Curcumin as a Drug

For nanocurcumin, not only their chemical composition but also their physical properties determine their characteristics. Physical and chemical properties are playing a major role in the alteration of normal curcumin into the nanoform (**Figure 3**). Particle size, surface area, surface charge, and hydrophobicity are important physicochemical properties that make nanocurcumin effective than native curcumin. Previous studies demonstrated that these properties can lead to an increased rate of solubility and higher oral bioavailability, including high pharmacokinetic profile, and active targeting (Biswas et al., 2014). Characteristics of curcumin vary with particle size change in the nanoscale. It was found that particle size reduction considerably improves the effectiveness of nanocurcumin and makes it superior to native curcumin. Mostly, 10–100 nm size nanoparticles have been used for various medicinal applications and clinical trials (Flora et al., 2013). Owing to its size, nanocurcumin is considered as an ideal choice to use as a drug compares to normal curcumin because of its larger surface area. Nanocurcumin enters organs that are almost cannot able to enter by curcumin. It was found that nanocurcumin may have a higher intracellular absorption capacity compared to normal curcumin. This property is also important to target intracellular pathogens for infectious diseases (Flora et al., 2013). It has been seen that nanocurcumin contains high systemic bioavailability in plasma and tissues compared to free curcumin (Zou et al., 2015). As per Ma et al. there is an increase in the *in vivo* bioavailability and distribution of the tissues due to nanocurcumin which offers 60 folds of increase in the biological half-life when compared as to the treatment of native curcumin in an experiment with rat models (Ma et al., 2007). Dende et al. reported nanocurcumin is better bioavailability than native curcumin and obstructing degenerative changes in cerebral malaria studies. There have been three folds of increase in the concentration of curcumin in the tissues of the brain when an oral dose of 5 mg PLGA-curcumin with 350 μg of curcumin was delivered than that accumulated with 5 mg of native curcumin (Dende et al., 2017). It was also found that nanoformulation of curcumin strengthens its circulation time, retention time, and mean residence time inside the body (Mythri et al., 2007). The surface area is also a paramount feature of nanoparticles. Primarily, materials made up of nanoparticles have a relatively larger surface area, and it increases the rate of degradation and aqueous solubility, which leads to enrichment of the bioavailability of drugs. Nevertheless, a large surface area enhances a drug response to a specific molecular target and improves its pharmacological activity (Mohanty and Sahoo, 2010). Due to the larger surface area, the drug injected into nanoparticles will be exposed to the particle surface encouraging to fast drug release. Also, the large surface area makes nanoparticles distinctive and suitable applicants for various applications. Brunauer–Emmett–Teller (BET) theorem is the simple and best method to determine the surface area of nanoparticle materials.

The role of the surface charges is established in curcumin nanoparticles. In general, the electric potential for the nanoparticles defines by surface charge, and it is completely related to nanoparticles chemical composition. Muller and Keck found that negative and positive zeta potential prevents the aggregation of nanoparticles. Thus, nanoparticles are extremely stable in suspension. Curcumin is forming aggregates and susceptible to opsonization because of its low solubility in water, while nanocurcumin dissolves completely in aqueous media forming no aggregates due to the presence of zeta potential (Muller and Keck, 2004). The positive charge obtained on the surface of nanoparticles is always considered being perfect because it can enter deep into cell membranes and have a high absorption rate compare to negatively charged particles. Also, nanoparticles along with a slight positive charge to improve its internalization capacity while a higher positive charge leads the toxicity to cells (Yallapu et al., 2015). On the other hand, the negative charge does not enter the cell wall at all but prevents it from breaking down under certain conditions and promotes a particle’s stability in circulation. No et al. described a relationship between surface charge and antimicrobial activity of nanocurcumin. Experimental data confirmed that positively charged curcumin nanoparticles showed better antimicrobial activity against *Listeria monocytogenes* (No et al., 2017).

Many biological processes such as protein adsorption and denaturation (Gessner et al., 2000), activation of immune cells, interaction with biological membranes or cellular uptake, and higher toxicity depend on the surface hydrophobicity (Chompoosor et al., 2010). Previous studies showed that the hydrophobicity of nanomaterials had a direct influence on the stability and bio-distribution of nanocarriers (Gessner et al., 2000; Jones et al., 2014). Therefore, it is a major object being controlled in the drug delivery systems. Due to hydrophobic nature,curcumin struggles to reach the cell membrane and bind through hydrogen bonding and hydrophobic interactions to the fatty acyl chains of membrane lipids. Thus, curcumin present inside the cytoplasm is very low. Nanoformulations of curcumin hold promise as a drug delivery system and overwhelmed these difficulties and increased its bioavailability. Loading efficiency and entrapment efficiency of nanodrug are highly depend on the preparation method and type of carrier system used to produce nanodrugs. Both play a vital role in drug delivery and had a great impact on the amount and level of drug release from the carrier. When loading efficiency is associated with the ratio of the drug to that of the carrier system, the entrapment efficiency tells of how much percentage of the drug in the nanoparticles that are being efficiently adsorbed or entrapped (Prokop and Davidson, 2008).

## Nanocurcumin Therapeutic Applications

Nanocurcumin is a promising therapeutic candidate with useful therapeutic properties viz., anti-inflammatory, anticancer, antiamyloid, antioxidant, antimicrobial antifibrosis and it has potential in the prevention and treatment of many human diseases. In the following section successful therapeutic applications of nanocurcumin are discussed.

### Anti-Inflammatory Effects

Curcumin is a potential anti-inflammatory agent and its anti-inflammatory activities mediated by the obstruction of enzymes activity, cytokines production, and activation of transcription factors. Wang et al. synthesized the curcumin-solid lipid nanoparticles (C-SLNs) and enhanced their effectiveness in an allergic rat model of asthma caused by ovalbumin. Experimental results revealed that airway hyperresponsiveness and inflammatory cell inﬁltration suppressed by C-SLNs. Also, C-SLNs mainly obstructed the expression of T-helper-2-type cytokines (interleukin-4 and 13) in bronchoalveolar lavage fluid (Wang et al., 2012). Milano et al. found that nanocurcumin is effective against esophageal adenocarcinoma (EAC) cell lines, OE33 and OE19. It sensitizes EAC cells to T cell-induced cytotoxicity and decreases the pro-inflammatory signals from T cells (Milano et al., 2013).

CUR-SLNs has enhanced solubility compared to its native form and significantly downregulated LPS-induced pro-inflammatory mediators (i.e., NO, PGE_2_, and IL-6) through obstructing the activation of NF-*κ*B in RAW 264.7 murine macrophage (Nahar et al., 2015). Similarly, nanocurcumin enhances oral bioavailability and thus increases effectiveness over to native form in the prevention of streptozotocin (ST) induced diabetes in rats, at least partly, by the suppression of inflammation and pancreatic beta-cell apoptosis (Ganugula et al., 2017). In another report, it was seen that loss of NF-κβ activation leads to the down-regulation of COX-2 and iNOS expression, obstructing the inflammatory response and tumorigenesis. The experimental study demonstrated that the curcumin-loaded PLGA nanoparticles (CUR-NP) decrease the pro-inflammatory mediators in *Staphylococcus aureus* affected mammary tissues *via* increasing NF-κβ signaling. Also, over to native curcumin, CUR-NP seems to be a good substitute against murine mastitis (Suresh et al., 2018). Hosseini et al. showed that the encapsulation of curcumin in nanomicelle had more anti-inflammation activity than curcumin to prevent the development of paraquat (PQ) induced lung injury (Hosseini et al., 2019). A recent investigation from Sinjari et al. gave evidence that the anti-inflammatory effect of curcumin liposomal formulations (CurLIP) in response to 2-hydroxyethyl methacrylate (HEMA) treatment in human dental pulp stem cells improved the quality of dental care with a major human community impact (Sinjari et al., 2019).

### Anticancer Effects

Many researchers have demonstrated the anticancer activity of curcumin on humans. It acts as a potential agent against human lung, breast, prostate, colorectal, liver, carcinoma, pancreatic, myeloma, and melanoma cancers due to the capability of inducing apoptosis, preventing cancer cell growth and suppression of cell cycle development (Shishodia et al., 2005). It was seen that curcumin prevents the growth of metastasis of cancer cells. Curcumin averts the attack of cancer cells in the normal tissue by obstructing the activity of matrix metalloproteinases that regulate the process. Curcumin suppresses the expression of genes cyclin D1, c-myc, bcl-2, Bcl-xL that are involved in tumor growth, proliferation, and apoptosis. For instance, the inhibition of nuclear factor-kappa (NF-κB) is important in carcinogenesis and proliferation. Curcumin deters the NF-κB activity that can increase the expression of genes related to proliferation (e.g., cyclin D1, c-myc), invasion (e.g., matrix metalloproteinases) and antiapoptotic (Tan and Norhaizan, 2019). Over to native curcumin, curcumin encapsulated in monomethoxy poly(ethylene glycol)-poly(3-caprolactone) (MPEG-PCL) micelles hindered the proliferation of 26 colon carcinoma at *in vivo* condition (Gou et al., 2011). Chen et al. synthesized the curcumin-loaded liposomes nanoparticles (CLNP) and then examined for the anticancer activity in B16BL6 melanoma cells. It revealed that the proliferation activity of the B16BL6 melanoma cells severely hindered by CLNP. It was mainly due to better drug delivery enabled by the fusion of particles and cell membranes of the lipids in the intracellular region. It also inhibits the PI3 K/AKT pathway that had a major role in skin carcinogenesis (Chen et al., 2012).

Basniwal et al. studied the effect of anticancer properties of curcumin nanoparticles in the lung (A549), liver (HepG2), and skin (A431) cancer cell lines. It was seen that curcumin nanoparticles showed a much better effect on the cancer cells compared to native curcumin at aqueous conditions (Basniwal et al., 2014). In another research, it has been demonstrated that PLGA-curcumin nanoparticles enhanced the lysosomal activity, apoptosis, inhibition of androgen receptor (AR), and nuclear β-catenin activity that resulted from a growth obstruction in prostate cancer cells (Yallapu et al., 2015). Triple-negative breast cancer (TNBC) is one of the most important histological subtypes of breast cancers having a metastatic phenotype. It has been demonstrated that dendrosomal nanocurcumin and exogenous p53 can act together to produce anticancer effects against TNBC cells (Baghi et al., 2018). HIF-1 and NF-κB are both indispensable for the improvement of cancer cell progression. PLGA nanoparticles (NP), loaded with curcumin (cur-PLGA-NP) elevated the HIF-1 and NF-κB subunits (HIF-1α and nuclear p65 (Rel A) expression in breast and lung cancer cells at the hypoxic microenvironment (Khan et al., 2018).

### Antiamyloid Effects

Amyloid beta (Aβ) is an important component associated with Alzheimer’s disease (AD). Thus, inhibition of the amyloid β‐peptide (Aβ) activities such as amyloid β‐peptide (Aβ) accretion, development of β‐amyloid fibrils (fAβ) from Aβ and the weakening of particular fAβ in the central nervous system offers probable targets for the treatment of AD. Several studies showed that curcumin regulated Aβ metabolism and inhibits Aβ aggregation in many ways to produce strong anti‐amyloidogenic effects against AD (Ohno et al., 2004). Cheng et al. developed a highly stabilized curcumin nanoparticle and orally administered to Alzheimer’s disease model, Tg2576 mice for 3 months. In comparison to native curcumin, nanocurcumin showed potent anti‐amyloidogenic effects in Tg2576 with reduced amyloid plaque density and improved bioavailability (Cheng et al., 2010). Apolipoprotein E3-mediated poly(butyl) cyanoacrylate nanoparticles containing curcumin (ApoE3-C-PBCA) effectiveness examined against β-amyloid SH-SY5Y neuroblastoma cells under *in vitro* condition. It revealed that ApoE3-C-PBCA had potent anti-amyloidogenic activity over the free form of curcumin and possible in the treatment of β-amyloid-induced cytotoxicity (Mulik et al., 2009). An investigation conducted by Mathew et al. described that conjugation of Tet-1 peptide to curcumin-PLGA nanoparticles showed the anti-amyloid effect against AD. It was seen that formulated curcumin had a strong affinity toward neurons by easily crossing the blood-brain barrier, and it has assisted the better obliteration of the amyloid aggregates, exhibiting its capability to treat AD (Mathew et al., 2012). Tiwari et al. reported curcumin encapsulated PLGA nanoparticles (Cur-PLGA-NPs) increased the anti-amyloid effect against AD in the rat model. Cur-PLGA-NPs enhanced the neuronal difference by triggering the Wnt/β-catenin pathway, related to the control of neurogenesis and can provide a therapeutic method to diagnosing AD, through improving a brain self-repair mechanism. In another study, solid lipid curcumin particles (SLCP) shown potent anti-amyloid effects in 5xFAD mice brain (Tiwari et al., 2014). Inhibition of Aβ42 activity through SLCP reduced the amyloid plaque load and decreased the irregular neuronal morphology in 5xFAD mice brain over to free curcumin (Maiti et al., 2018).

### Antioxidant Effects

Curcumin’s antioxidant activity has been revealed in biological models by many researchers. There are so many scientific evidence on the capability of curcumin on living cells in trapping the free radicals like reactive nitrogen and oxygen species through several means and thus exhibiting the antioxidant property (Rafiee et al., 2019). It was demonstrated that curcumin increases the efficacy of free radicals scavenging activity related enzymes, but also produces inhibitory effects on enzymes which produce free radicals (Hewlings and Kalman, 2017). Curcumin nanoparticles (CURN) prepared by Yen et al. using a simple nanoprecipitation technique with polyvinylpyrrolidone (PVP) as the hydrophilic carrier (Yen et al., 2010). CURN displayed the strong free radical scavenging activity and improved anti-lipid peroxidation effect than a native complement to human hepatoma cell lines HepG2, PLC/PRF/5, and Hep3B. Kakkar et al. evaluated the neuroprotective potential of curcumin loaded solid lipid nanoparticles (C-SLNs) in bilateral common carotid artery occlusion (BCCAO) induced global cerebral ischemia (GCI) in rats. The antioxidant activity can be increased by enhancing the bioavailability of C-SLNs and the effective mobilization of a cerebral ischemic insult. This also inhibits the effects over the conversion of xanthine dehydrogenase/oxidase and effects in the resulting of superoxide anion (Kakkar et al., 2013).

Alginate–curcumin nanoparticles (Alg-NP-Cur) were prepared and examined against Parkinson’s disease in the drosophila model. Alg-NP-Cur displayed effective antioxidant activity through the decrease of the lipid peroxidation in the Parkinson’s disease drosophila brain after a diet supplemented with the nanocarrier for 24 days (Siddique et al., 2013). Curcumin nanocrystals used its antioxidant effect for reducing lipid peroxidation, and by improving the activities of antioxidant and detoxification enzymes against circulatory toxicity in Wistar rats (Rajasekar, 2015). Moghaddasi et al. explained the synthesis of the nanocurcumin system (nano-CUR) using the O/W nanoemulsion method. The antioxidant activities of nano-CUR have more potential than its native curcumin and *in vitro* cytotoxicity eﬀect of nano-CUR was examined in Neuro2A cells suggests that nano-CUR has the potent candidate for the treatment of chronic diseases (Moghaddasi et al., 2018). In another research, Ranjbar et al. studied the curcumin and nano-curcumin effects on the oxidant and antioxidant system on the liver mitochondria using aluminum phosphide (AIP) toxicity induced rat model. It was seen that nanocurcumin enhanced the oxidative stress factors and protected the liver against the adverse effects of AlP through the scavenging of free radicals and stabilizing the oxidative status (Ranjbar et al., 2020).

### Antimicrobial Effects

Curcumin’s antimicrobial activity mechanism is strongly linked to the interaction with the FtsZ protein inducing cell division. Reports conclude that curcumin’s methoxy and hydroxyl groups are straightly linked to the antimicrobial activity (Han and Yang, 2005). According to Kaur et al. all the oxygen molecules of phenol, two carbonyl groups, and methoxyl functional groups that are linked to phenolic rings of curcumin are entangled in hydrophobic-hydrogen bonds along with FtsZ GTPase. These moieties catalyze the protein FtsZ GTPase and thus leading to the death of the cancer cells (Kaur et al., 2010). Like curcumin, nanoformulated curcumin’s antimicrobial activity against a wide range of microorganisms including fungi, bacteria, and viruses has been described by many researchers. Nanocurcumin exhibits improved antibacterial activity than curcumin because of its enhanced aqueous-phase solubility and simple dispersibility. The efficient antibacterial activity was seen against *Bacillus subtilis*, *S. aureus, Helicobacter pylori*, and *Pseudomonas aeruginosa* (Basniwal et al., 2011). It was found that silver-decorated polymeric micelles encapsulated with curcumin exhibited strong antibacterial activity to *P. aeruginosa* and *Staphylococcus aures* (Huang et al., 2017). Similarly, Zaharieva et al. reported that curcumin loaded micelles enhance the alkylphosphocholines erufosine and miltefosine antibacterial activities against pathogenic *S. aureus* strain (Zaharieva et al., 2019). Wang et al. demonstrated that encapsulated curcumin exhibited a broad spectrum of antifungal activity by obstructing *S. cerevisiae*, *Aspergillus niger*, and *Penicillium notatum*. Hydroxyl propyl methyl cellulose and polyvinyl pyrrolidone successfully used to prepare the curcumin hydrogel nanoparticles (Wang et al., 2009). It controls the parasites associated with the pathogenesis of malaria (Dandekar et al., 2010).

Gandapu et al. demonstrated that curcumin-loaded apo transferrin nanoparticles hindering the HIV multiplication by its capacity to target the endocytosis-promoting cellular receptor. Nanoparticles exhibited continuous curcumin delivery and decreased cytotoxicity of curcumin up to 50% over to its free form. Moreover, curcumin nanoformulation exhibited three-times higher anti-HIV activity over to its free form and obstructed the HIV-1 caused expression of IL-1β, Topo II α, and COX-2 and entirely stopped the synthesis of viral complementary DNA (cDNA) (Gandapu et al., 2011). In a similar manner, formulated curcumin such as curcumin modified silver nanoparticles (cAgNPs) is used to inhibit the respiratory syncytial virus (RSV) infection cells. It controlled the RSV infection and providing a reduced amount of viral loads with no toxic effect (Yang et al., 2016). One more research, Naseri et al. investigated the anti-viral effects of curcumin nanomicelles on the attachment and entry of hepatitis C virus (HCV) infection. It was seen that viral load for HCV cells treated with curcumin nanomicelles was decreased (Naseri et al., 2017).

### Antifibrosis Effects

Many researchers studied and confirmed that curcumin is a better option for the treatment of fibrosis. It can obstruct the development of fibrosis by attenuating the expression of cytokines and chemokine genes that directly involved in the fibrosis and the initiation of apoptosis in stellate cells of affected organs. Bisht et al. used nanocurcumin to treat animals with hepatic injury and fibrosis induced by carbon tetrachloride (CCl_4)_ administration under *in vivo* studies. It was seen that nanocurcumin can able to improve the activity of CCl_4_-induced liver injury and the following fibrosis in rodents. Such results are correlated with inhibiting the development of pro-inflammatory cytokines, increasing intrahepatic antioxidant rates and decreasing profibrogenic transcripts. Also, nanocurcumin hinders profibrogenic transcripts linked with triggered myofibroblasts and straight induces apoptosis (Bisht et al., 2011). Similarly, Son et al. experimental results revealed that nanocurcumin has significant effects on decreasing levels of serum alanine aminotransferase (ALT) and aspartate aminotransferase (AST) in CCl4-induced hepatic fibrosis mice. Histopathological evaluation revealed that hepatic fibrotic livers of mice treated with nanocurcumin were recovered after 4 weeks (Son et al., 2013). In another study, Alvarino and Yanwirasti confirm that nanocurcumin supplementation was able to attenuate MMP-9 expression in rat’s kidney that suffers unilateral ureter obstruction (UUO). It was reduced fibrosis area in interstitial and tubular atrophy of rat’s kidney that suffers UUO (Alvarino and Yanwirasti, 2018).

### Other Biological Effects

A research group tested and confirmed the anticonvulsant effect of liposome entrapped curcumin. It enhanced the current electroshock seizures (ICES) and pentylenetetrazole (PTZ)-induced seizures and status epilepticus in mice (Agarwal et al., 2011). Encapsulated curcumin nanoparticles (ECNPs) therapeutic effects against arsenic-induced toxicity in rats was demonstrated by Yadav et al. It was found that ECNPs had an abundant distinct effect in reversing the opposite changes that appeared due to oxidative stress generated through arsenic (Yadav et al., 2012). Besides, ECNP had a strong chelating effect at a low dose (1.5 mg/kg) compared to unformulated curcumin. Curcumin found to be useful for the treatment of normal and diabetic-impaired wounds (Jagetia and Rajanikant, 2012; Mohanty et al., 2012; Kulac et al., 2013; Kant et al., 2014). Merrell et al. developed the curcumin‐loaded poly(caprolactone) (PCL) nanofibers matrix and evaluated its effect in human foreskin fibroblast cells (HFF‐1) through oxygen radical absorbance capacity (ORAC) assay. HFF-1 displayed more than 70% viability and suggest that curcumin‐loaded nanofibers potent wound healing agent (Merrell et al., 2009). Krausz et al. prepared the curcumin loaded silane-hydrogel nanoparticle vehicle (curc-np) and investigated the bioavailability and potential of wound healing activity. It was found that curc-np hindered the *in-vitro* growth of methicillin-resistant *S. aureus* (MRSA), and arrested the MRSA growth and showed better wound healing activity in murine wound model (Krausz et al., 2015). In another research, curcumin chitosan nanoparticles (CSNPs) loaded nanohybrid scaffold was showed a potent effect against diabetic wounds (Karri et al., 2016). Bajpai et al. reported that cellulose nanocrystals (CNC) together with chitosan to produce a dressing film and then encapsulated with curcumin and silver nanoparticles, exhibit better-wound healing activity in albino Wistar rats (Bajpai et al., 2017).

## Clinical Trials and Patents

So far, many clinical trials have explained the pharmacokinetic profile, safety, and effectiveness of curcumin to different diseases. Clinical trials showed some positive results that curcumin arrests or even eliminate the growth of cancer cells. To date, a total of 210 clinical trials related to curcumin were listed in the United States National Library of Medicine (clinicaltrials.gov). Among them, 92 clinical trials were completed and 32 clinical trials status is unknown, while reminder was recruiting, active/not recruiting, suspended, terminated, completed, and withdrawn. Several clinical trials demonstrated the effectiveness of nanocurcumin in cancer, multiple sclerosis, amyotrophic lateral sclerosis, ankylosing spondylitis, chronic kidney disease, and metabolic syndrome patients. Some nanocurcumin clinical trials were given in **Table 3**. Several clinical trials also published. Recently, Ahmadi et al. conducted a clinical trial and showed that nanocurcumin is a safe and effective treatment in patients with amyotrophic lateral sclerosis (Ahmadi et al., 2018). Another clinical trial conducted by Dolati et al. suggested that nanocurcumin is capable of restoring the frequency and function of treg cells in multiple sclerosis patients (Dolati et al., 2019).

**Table 3:** Details of clinical trials conducted with curcumin nanoformulations.

S. No	Clinical trials.gov identifier	Study title	Status	Applications against disease	Responsible for investigation
1	NCT01403545	Evaluation of liposomal curcumin in healthy volunteers	Completed	Drug safety	Medical University of Vienna, Vienna, Austria
2	NCT01925547	Micellar curcumin and metabolic syndrome biomarkers	Completed	Metabolic syndrome	University of Hohenheim, Germany
3	NCT01201694	Study on surface controlled water soluble curcumin	Completed	Cancer	UT MD Anderson Cancer Center Houston, Texas, United States
4	NCT03150966	The immunomodulatory effects of oral nanocurcumin in multiple sclerosis patients	Completed	Multiple sclerosis	Tabriz University of Medical Sciences, Iran
5	NCT03140657	The effects of nanocurcumin on treg cells and Th17 cells responses in ankylosing spondylitis patients	Completed	Ankylosing spondylitis	Tabriz University of Medical Sciences, Iran
6	NCT01982734	Improved oral bioavailability of curcumin incorporated into micelles	Completed	Drug safety	University of Hohenheim, Germany
7	NCT03534024	The effects of nanomicelles curcumin on glycemic control, serum lipid profile, blood pressure, and anthropometric measurements in patients with metabolic syndrome	Recruiting	Metabolic syndrome	National Nutrition And Food Technology Research Institute, Iran
8	NCT03514667	The effects of nanocurcumin on serum oxidative stress inflammation, adiponectin, and NF-kB in blood mononuclear cells in metabolic syndrome patients (Nuclear Factor-κB)	Recruiting	Metabolic syndrome	National Nutrition and Food Technology Research Institute, Tehran, Iran
9	NCT01294072	Study investigating the ability of plant exosomes to deliver curcumin to normal and colon cancer tissue	Active, not recruiting	Colon cancer tissue	University of Louisville, United States
10	NCT02724618	Nanocurcumin for prostate cancer patients undergoing radiotherapy (RT)	Active, not recruiting	Prostate cancer	Shahid Beheshti University of Medical Sciences, Iran
11	NCT01001637	Efficacy and safety of curcumin formulation in Alzheimer’s disease	Unknown	Alzheimer’s disease	Jaslok Hospital and Research Centre, Maharashtra, India
12	NCT02683759	Bio-enhanced curcumin as an add-on treatment in maintaining remission of ulcerative colitis	Unknown	Ulcerative colitis	Asian Institute of Gastroenterology, Hyderabad, India

Source: Clinical trials information obtained from U.S. National Library of Medicine clinical trial (https://clinicaltrials.gov/) website

Several curcumin nanoformulation patents include liposomal curcumin (Kurzrock et al., 2011), chitosan nanoparticles encapsulated curcumin (Kumar et al., 2012), polymer nanoparticles loaded curcumin (Braden and Vishwanatha, 2008; Ranjan et al., 2010), oil emulsion of curcumin (Khamar et al., 2013), vesicles loaded curcumin (Shen et al., 2012), antioxidant nanoemulsions of curcumin (Pathak and Tran, 2012), curcumin cyclodextrin (Yallapu et al., 2010), glycyrrhetinic acid-mediated curcumin long-circulating nanostructured lipid carrier (Li, 2017), curumin bound to fibroin polypeptide and curcumin loaded magnetic nanoparticles, and acidic sophorolipid encapsulated curcumin (Chauhan et al., 2015) have been made. Several registered patents on curcumin nanoformulations summarized in **Table 4**. The patent of WO2009105278A2 described the preparation of curcumin encapsulated chitosan nanoparticles by ionotropic gelation method and delivery into extra-testicular Sertoli cells. The patent reported almost all of the delivered curcumin in the Sertoli cells was spread all over the lungs (Kumar et al., 2009). The discovery of US patent US 8535693 B2 involving the treatment of inflammation, skin, and mucosal disorders by topical nanoparticles. Curcumin and emulsifier/nonionic surfactant mixture synthesized as nanoparticles by sonication. It was topically tested in mice and seen that a continuous granular layer and a good epidermal thickness with the treatment of the formulated curcumin (Chaniyilparampu et al., 2010). Santhosh Kumar et al. developed the curcumin chitosan nanoparticles and tested the bioavailability of curcumin in mice (WO2010013224A2). It was found that a 10-fold increase of curcumin along with long time circulation over native curcumin (Santosh Kumar et al., 2010).

**Table 4:** Details of registered patents on curcumin nanoformulation.

S. No	Title of the patent	Patent/application number	Reference
1	Nanoparticle targeted drug delivery to the lungs using extra-testicular Sertoli cells	WO2009105278A2	Kumar et al., 2009
2	Topical formulation(s) for the treatment of inflammation, skin and mucosal disorders, and other diseases	US 8535693 B2	Chaniyilparampu et al., 2010
3	Curcumin nanoparticles with improved bioavailability and methods of producing the same patent	WO2010013224A2	Santosh Kumar et al., 2010
4	Preparation method and application of curcumin chitosan-stearic acid graft micelle	CN102743336A	Xianwang et al., 2012
5	Magnetic nanoparticle formulations, methods for making such formulations, and methods for their use	US 20130245357Al	Chauhan et al., 2013
6	Nanocrystalline solid dispersion compositions and process of preparation	WO 2013132457 A2	Bansal et al., 2013
7	Curcumin coated magnetite nanoparticles for biomedical applications	WO2013108270A1	Pattayil and Jayphraba, 2013
8	Nanoparticles for mitochondrial trafficking of agents	WO 2013123298 A1	Dhar and Marrache, 2013
9	Curcumin-er, a liposomal-PLGA sustained release nanocurcumin for minimizing qt prolongation for cancer therapy	US 20140065061A1	Ranjan et al., 2014
12	Novel highly bioavailable, water soluble and sustained release nanoformulations hydrophobic plant derived compounds and extracts	US 20150072012 A1	Sripathy et al., 2015
13	Nanomicelles for the treatment of cancer	WO2016167730A1	Oguz et al, 2016
14	Curcumin-sophorolipid complex	WO2016013026A1	Singh et al., 2016
15	Curcumin long-circulating nanoliposome carrier of enoxolone mediation and preparation method	CN104689321B	Li, 2017
16	Phospholipid/chitosan drug delivery system, preparation method, and uses	WO2017186065A1	Liu et al., 2017
17	Production of curcumin and piperine loaded double-layered biopolymer based nano-delivery systems by using electrospray/coating method	EP3142702B1	Sezgin and Bayraktar, 2018

Source: Curcumin nanoformulation patents information obtained from Google Patents website

In the patent CN102743336A (Xianwang et al., 2012), preparation method and application of a curcumin chitosan-stearic acid (CSO-SA/curcumin) graft micelle was detailed in cancer cells. In vivo studies showed that CSO-SA/curcumin can able to kill the efficacy of MCF-7, MCF-7/Adr, and colorectal cancer cells without any toxic effects. Curcumin loaded magnetic nanoparticles induced apoptosis in cancer cells. Experimental data from this study showed improved bioavailability compared to native curcumin in the mouse. Also, obstruction of pancreatic tumor growth was observed in mouse treated with nanocurcumin. This invention registered in the US patent US 20130245357A1 (Chauhan et al., 2013). Bansal et al. developed the unique nanocrystalline solid dispersion composition to release the curcumin into the intestine and registered the patent WO 2013132457 A2. Dry powder of curcumin: stearic acid (nanocrystalline solid dispersion) prepared and tested in rats. It revealed improvement of curcumin bioavailability 15-folds compared to normal curcumin (Bansal et al., 2013). The invention WO2013108270A1 from Pattayil and Jayaphraba developed the curcumin coated ultra-small super paramagnetic iron oxide nanoparticles (USPION) for biomedical applications. The authors showed the synthesis of biocompatible and stable curcumin by the simple one-pot process (Pattayil and Jayaphraba, 2013).

The patent WO 2013123298 A1 discloses the synthesis of nanoparticles with a mitochondrial targeting moiety. Dhar and Marrache prepared an eco-friendly biodegradable polymer with a terminal OH group (PLGA-b-PEG-OH) to permit the conjugation of triphenylphosphonium (TPP), thus obtaining PLGA-b-PEG-TPP. Curcumin encapsulated nanoparticles were synthesized through the nanoprecipitation method and their ability was evaluated in neurodegenerative diseases. It was seen that better neuroprotection with curcumin nanoparticles over native curcumin against β-amyloid plaques (Dhar and Marrache, 2013). The patent US 20140065061A1 discloses hybrid curcumin nanoformulation based on liposome PLGA prepared by the emulsification method. This formulation improved the bioavailability and declining qt prolongation in cancer therapy (Ranjan et al., 2014). The invention of European patent EP2649623B involves curcumin loaded magnetic nanoparticles described the effectiveness in various cancer cells (Chauhan et al., 2015). Curcumin nanoformulation named CurQLife^®^ synthesized by adding curcumin into a pre-heated solution containing polyethylene glycol (PEG) 200 and Tween 20 (US 20150072012 A1) (Sripathy et al., 2015). *In vivo*, experimental results on rats and humans showed a better bioavailability of CurQLife ^®^ in comparison with other bioavailable curcuminoid products in the market.

The invention of patent WO2016167730A1 described the curcumin nanoparticles and their effectiveness for the treatment of cancer (Oguz et al., 2016). The patent of WO2016013026A1 consisting of acidic sophorolipid and curcumin [SL (A) +Cur], in which, curcumin is solubilized and nano-encapsulated in acidic sophorolipid to improve curcumin’s bioavailability and solubility to increase its therapeutic activity including cancer (Singh et al., 2016). In the patent of CN104689321B, the preparation of glycyrrhetinic acid-mediated curcumin long-circulating nanostructured lipid carrier dispersion liquid was reported. This nanolipid carrier consists of glycyrrhetinic acid-phospholipid derivative, soybean lecithin and polyoxyethylene 40 stearate, caprylic/capric triglyceride, and glyceryl monostearate (Li, 2017). Liu et al. registered the patent of WO2017186065A1 curcumin delivery system based on nanoparticles such as phospholipid and chitosan (Liu et al., 2017). Recently, European patent EP3142702B1 describes the preparation of curcumin and piperine loaded biopolymer-based nano-delivery systems using electrospray/coating techniques with improved curcumin bioavailability (Sezgin Veliddin and Bayraktar, 2018).

## Research Gap and Future Perspectives

Curcumin has been received broad attention over the decades for its potential therapeutic applications. With in-depth review of the literature, it is worth mentioning that nanoencapsulation techniques enhanced the pharmacokinetic properties of the drug packed with curcumin and offered better therapeutic value. In the various sections of this review, according to the content mentioned, numerous curcumin nanoformulation has been developed and used to treat many diseases in human as well as enormous progress has been achieved by curcumin nanoformulation over the past decades. However, the dictum “there is always room for improvement” is precisely in agreement with the pace of the ongoing developments to make curcumin as an effective drug candidate. Hence, many challenges and questions still exist to propose nanocurcumin as a promising candidate for therapeutic applications in human diseases. So far, numerous curcumin nanoformulations have been introduced to improve the cellular uptake, tissue specificity, and effectiveness of curcumin. In this review, some of the curcumin nanoformulations discussed potently challenge many signaling pathways that are linked to various human diseases. Most of these formulations, however, remained at the proof of concept stage and experiments were performed only in the pre-clinical models, and therefore our lack of understanding of the risks of curcumin nanoformulation in humans is a major issue. Thus, always question the toxicological safety of curcumin applications. Unfavorable toxicity arising through the nanomedicine based drug delivery methods result in DNA damage, allergic responses, neuroinflammation, and excitotoxicity. For this peculiar reason, the biocompatibility and biodegradability of the nanomedicines have to be researched and recorded with accuracy.

So far, very limited clinical studies only conducted, they confirm that nanocurcumin has better characteristics such as bioavailability, chelating property, and retention time compare to bulk curcumin as well as systematically safe. However, substantial gaps in research have been identified due to the limited number of clinical trials to assess the safety and efficacy of curcumin nanoformulations in humans. Thus, it is necessary to conduct the many clinical trials with a large group of patients before introducing the curcumin nanoformulations to the pharmaceutical market. Curcumin nanoparticles are not tissue specific and in this sense, they are just delivered onto the healthy tissues found around the tumor or cancer cells. So, larger attention may be focused on the development of nanodrug delivery systems that could be tissue-specific. Hybrid nanoparticles (comprised of two or more components comprised each other enveloped curcumin) developed for a specific cell targeting. These hybrid nanoparticles exhibit potent cytotoxicity in cancerous cells compared with nanoparticles and free curcumin. However, further human considerations are required to evaluate the efficacy and toxicity of hybrid nanoparticles with clinical trials.

It is also worth investigating that whether curcumin can be used as a drug alone or in a suitable formulation with an additional drug, which could enhance its potential for the frontiers of chemotherapeutic strategies is yet to be addressed. In this view, curcumin-loaded nanoparticles should be incorporated into any other therapeutic treatment to reduce the amount of the main drug which can give the outcome of improved therapeutic activities with less toxicity. As a result, it can improve the therapeutic efficacy of curcumin-loaded nanoparticles along with less toxicity. Although, researchers should give priority to expanding the industrial production of nano-encapsulated curcumin. For this reason, discovering cost-effective techniques to nanoencapsulate curcumin is an industrial requirement for decreasing manufacture prices and open outstanding competition with synthetic additives and drugs. Finally, the application of nanocurcumin is still in its initial phases. Its progress requires serious and committed efforts through a system of organized and scheduled trials based entirely on the goal of enhancing curcumin’s beneficial effects.

## Author Contributions

AK built the layout of the article, collected literature, and wrote the article. TM and NS provided suggestions in manuscript writing. AK and TM edited it.

## Conflict of Interest

The authors declare that the research was conducted in the absence of any commercial or financial relationships that could be construed as potential conflict of interest.
